# Sustaining Fragile Gains: The Need to Maintain Coverage with Long-Lasting Insecticidal Nets for Malaria Control and Likely Implications of Not Doing So

**DOI:** 10.1371/journal.pone.0083816

**Published:** 2013-12-27

**Authors:** Lucy Smith Paintain, Jan Kolaczinski, Melanie Renshaw, Scott Filler, Albert Kilian, Jayne Webster, Kojo Lokko, Matthew Lynch

**Affiliations:** 1 Department of Disease Control, London School of Hygiene and Tropical Medicine, London, United Kingdom; 2 Strategy, Investment and Impact Division, The Global Fund to Fight AIDS, Tuberculosis and Malaria, Geneva, Switzerland; 3 African Leaders Malaria Alliance, Geneva, Switzerland; 4 Tropical Health LLP, Montagut, Girona, Spain; 5 Center for Communication Programs, Bloomberg School of Public Health, Johns Hopkins University, Baltimore, Maryland, United States of America; Tulane University School of Public Health and Tropical Medicine, United States of America

## Abstract

Global commitment to malaria control has greatly increased over the last decade. Long-lasting insecticidal nets (LLINs) have become a core intervention of national malaria control strategies and over 450 million nets were distributed in sub-Saharan Africa between 2008 and 2012. Despite the impressive gains made as a result of increased investment in to malaria control, such gains remain fragile. Existing funding commitments for LLINs in the pipeline to 2016 were collated for 40 sub-Saharan African countries. The population-based model NetCALC was used to estimate the potential LLIN coverage achievable with these commitments and identify remaining gaps, and the Lives Saved Tool (LiST) was used to estimate likely consequences for mortality impact if these gaps remain unfilled. Overall, countries calculated a total need of 806 million LLINs for 2013-16. Current funding commitments meet just over half of this need, leaving approximately 374 million LLINs unfunded, most of which are needed to maintain coverage in 2015 and 2016. An estimated additional 938,500 child lives (uncertainty range: 559,400–1,364,200) could be saved from 2013 through 2016 with existing funding (relative to 2009 LLIN coverage taken as the ‘baseline’ for this analysis); if the funding gap were closed this would increase to 1,180,500 lives saved (uncertainty range: 707,000–1,718,900). Overall, the funding gap equates to approximately 242,000 avoidable malaria-attributable deaths amongst under-fives. Substantial additional resources will need to be mobilized to meet the full LLIN need of sub-Saharan countries to maintain universal coverage. Unless these resources are mobilized, the impressive gains made to date will not be sustained and tens of thousands of avoidable child deaths will occur.

## Background

Global commitment to malaria control has greatly increased over the last decade, with an associated and unprecedented financial contribution, peaking at $2 billion per annum in 2011 [1]. Long-lasting insecticidal nets (LLINs) have become a core intervention of national malaria control strategies and over 450 million nets were distributed in sub-Saharan Africa between 2008 and 2012 (Data available from: http://www.allianceformalariaprevention.com/working-groups-view.php?id=19).

LLINs and their predecessors, insecticide-treated nets (ITNs) have a well-documented impact on child mortality [2]–[5]. The UN Secretary-General Ban Ki Moon issued a call on World Malaria Day 2008 for “universal coverage” of key malaria interventions, including LLINs, by 2010. A number of published studies have demonstrated significant improvements in malaria indicators since the year 2000 [6], and the dramatically increased funding of the last five years has allowed most national malaria control programmes to scale up malaria interventions. Despite the impressive gains made by the increased investment in malaria control, historical data demonstrate that such gains are fragile; a recent review found that the vast majority of examples of malaria resurgence could be attributed in large part to weakened investment in sustaining malaria control [7]. Brief resurgences of malaria incidence identified in Rwanda and Zambia in 2009 add further support to this conclusion [8]. It is therefore vital that these fragile gains are sustained, as it is unlikely that the current momentum and support to malaria control could be easily re-established. Indeed, political support for sustaining fragile gains was given at the 2012 United Nations General Assembly [9]. It is estimated that US$2.7 billion annually are required for sub-Saharan Africa to achieve and maintain universal coverage with core malaria prevention, diagnosis and treatment services [10]. Even at the funding peak of $2 billion in 2011, global resources for malaria control still fell short of this annual requirement. There is an urgent need to maintain advocacy for the health and economic benefits that investment in malaria control can achieve.

This paper collates existing funding commitments for LLINs for sub-Saharan Africa in the pipeline to 2016 and aims to estimate the potential coverage achievable, identify what gaps remain and estimate the potential consequences for mortality impact.

## Methods

### Sources of data on LLIN requirements and available funds

The numbers of LLINs needed and funded for the period 2013–2016 were obtained from individual country gap analyses by 40 malaria-endemic countries in sub-Saharan Africa. The development of the country gap analyses was supported by the Roll Back Malaria (RBM) Harmonisation Working Group (HWG) (http://www.rollbackmalaria.org/toolbox/tool_ProgrammaticGapAnalysis.html). Calculations of the number of LLINs needed were based on the goals and targets of each country’s national malaria strategic plans; however general guidelines were as follows:

- The population at risk of malaria was defined for each year, including projections of population growth.
- In the majority of countries, campaigns are planned to take place every three years. The number of LLINs required for a planned campaign in a given year was calculated as the population at risk divided by 1.8; this is based on an allocation of one LLIN per two persons, adjusted for the effect of odd-numbered households [11].
- The number of LLINs required for routine distribution to pregnant women and/or infants was calculated as the number of vulnerable individuals multiplied by the coverage of the delivery channel (for example, antenatal clinics and/or the expanded programme of immunisation); expected improvements in coverage of these delivery channels over time were incorporated as appropriate.
- The number of LLINs required for other continuous distribution channels (e.g. school- or community-based delivery) was calculated as the number of target individuals multiplied by the coverage of the delivery channel.
- If population coverage of LLINs through routine LLIN distribution is greater than 40%, existing LLINs were accounted for when calculating need for campaign nets using recommended rates of LLIN loss (8% one year post-distribution, 20% two years post-distribution, and 50% three years post-distribution) (http://www.rollbackmalaria.org/mechanisms/hwg.html). If population coverage through routine channels was less than 40%, existing nets were not accounted for in the calculations for campaign nets as recommended by the RBM vector control working group. This threshold is based on the practicalities and cost effectiveness of identifying existing nets during a mass campaign, and the age of those nets in terms of their likely condition and level of protection offered [12]. This approach of only accounting for existing routine nets above a certain threshold coverage explains why the country gap analyses identify a greater LLIN need than the WHO 2012 World Malaria Report estimates which do not include such a calculation.

Once a country had identified the number of LLINs required to meet their strategic plan, they mapped current funding from all donors and domestic financing. This included disbursed funds as well as best estimates based on commitments or previous funding levels. Specifically, this includes phase two funding from unsigned Global Fund grants, funds through the Global Fund transitional funding mechanism (TFM) and interim new funding modality, and projections of funding from bilateral donors such as the US government’s President’s Malaria Initiative (PMI), the World Bank and other donors.

It is important to note that obtaining the most up-to-date information on projections of LLINs in-country or in the pipeline is challenging and figures are dynamic. However, country-level data was assumed to be the most robust for the purposes of this analysis. More information on the gap analyses is available from http://www.rollbackmalaria.org/toolbox/tool_ProgrammaticGapAnalysis.html.

The number of LLINs distributed over the period 2010–2012 was taken from the Net Mapping Project, which collates country-level data from all of the WHOPES-approved LLIN manufacturers on a quarterly basis, considered to be the most objective measure of LLINs shipped to country (http://www.allianceformalariaprevention.com/working-groups-view.php?id=19). Since this analysis is concerned with predictions of LLIN coverage and impact, a three month delay was applied to each quarter’s data as a proxy for the delay between LLINs arriving in country and actually being distributed to recipients (File S1). It is important to note that timing of distribution was determined at country level and there may have been longer delays in some instances.

### Predicting LLIN coverage

Country level numbers of LLINs distributed, funded or needed were converted to predictions of household ownership of at least one LLIN and the proportion of the population with access to an LLIN using the NetCALC spreadsheet model (available at: http://www.networksmalaria.org/networks/netcalc). NetCALC is a simple deterministic cohort model that uses the timing and size of net distribution, population and household size, population growth, intra-household net accumulation, and net decay to estimate household and population net coverage levels over time.

In overview, NetCALC projects LLIN coverage at a given point in time based on the number of nets available for use; this is the sum of all nets remaining from annual net cohorts assuming an S-shaped loss function, based on field data of LLIN longevity [11], [13], [14] which is mathematically described allowing the user to select a variety of median survival times. The relationship between the mean number of nets per net-owning household and the mean number of nets per household considering all households was defined using several household surveys from Uganda at varying levels of net coverage. The relationship was modelled using a fractional polynomial regression and a correction factor added to account for variations in average household size from country to country. Thus, by inputting population parameters, expected median lifespan of LLINs, and numbers of LLINs distributed, NetCALC estimates household ownership and population coverage over a set period of time. More details of the model and its validation are provided in File S2. The tool has been used in previously published work [12], [13], [15].

For each of the 40 sub-Saharan African countries included in this analysis, the following data were collated for entry in to the NetCALC model:

- Total population and annual growth rate were taken from the UN Population Division database for 2010 [16].
- The proportion of population at risk of malaria was taken from the MARA mapping project [17]. At-risk populations covered by IRS in countries such as Eritrea, Madagascar and Mozambique were not accounted for in this analysis.
- Mean household size was extracted from recent nationally-representative household surveys where available (Demographic Health Surveys or Malaria Indicator Surveys available from: http://www.measuredhs.com/pubs/; or Multiple Indicator Cluster Surveys available from: http://www.childinfo.org/mics3.html). In the absence of recent survey data, the median of five persons per household was used.
- The year 2009 was taken to be the baseline for this analysis based on the assumption that most countries conducted their major scale-up activities over the period 2010-12. To account for ITNs already present from earlier distributions, nationally-representative survey data available for the year closest to 2009 was used as a ‘baseline’ household ITN ownership (with an additional assumption that 90% of these ITNs were LLINs). In the absence of survey data for 2009, published estimates of household ITN ownership were used for nine countries (Chad, Comoros, Djibouti, Eritrea, Ethiopia, Guinea, Mali, Sudan and Zimbabwe) [18].
- In line with current RBM guidelines, median LLIN lifespan was set at three years [1].
- The number of LLINs distributed between 2010 and 2012, and the number needed for 2013–2016 were entered to estimate the coverage (household ownership) that could be achieved if countries had sufficient funding to fully meet their calculated needs.
- Lastly, the number of LLINs actually funded for 2013–2016 were entered to estimate the household ownership that would be achieved if no further funding was mobilised to fully meet a country’s calculated needs.

### Predicting mortality impact

The Lives Saved Tool (LiST) was used to make predictions of the mortality impact of the estimates for household ITN ownership generated by NetCALC. LiST is a multi-cause model of mortality that uses country-specific data on causes of death, population size and growth, and best available evidence for the effectiveness of child and maternal health interventions to estimate the mortality impact of scaling up these interventions [19].

Standard country profiles were used as downloaded from the LiST website in January 2013 (http://www.jhsph.edu/departments/international-health/centers-and-institutes/institute-for-international-programs/list/). Lives saved per year due to the change in LLIN coverage compared to 2009 coverage were estimated based on the annual LLIN coverage predicted by NetCALC for years 2010 to 2016 if: (i) all LLINs were funded, or (ii) with currently funded LLINs. The difference between these two predictions is termed the ‘mortality gap’, meaning the potential number of children whose lives will not be saved if resources are not mobilised to fully fund countries’ identified LLIN needs.

The lives saved are those due to malaria only (i.e. are not all-cause deaths). Coverage of all other (non-ITN) interventions was kept constant for the period of LiST predictions (2013-2016), including treatment of malaria with an artemisinin compound. The following parameters are those that are critical for the predictions in this analysis:

- *Protective efficacy (PE) of ITNs.* In the baseline estimates, a PE of 0.55 was used to estimate the mortality impact of household ownership of at least one LLIN [20]; this is based on data from the original ITN trials and has been validated against country data [21]
- *The relative proportion of post-neonatal deaths (children aged 1-59 months) due to malaria in 2009.* This was obtained for each country in the analysis from the authors of the recent global causes of mortality figures [22].

Sensitivity analyses were conducted varying these two key parameters for each country. To estimate the maximum mortality impact achievable by LLINs, the upper confidence limit of the PE of ITNs calculated by *Eisele et al* (PE = 0.60) was combined with the upper uncertainty limit for the proportion of post-neonatal mortality due to malaria [20], [22]. To estimate the minimum mortality impact achievable by LLINs, the lower confidence limit of the PE of ITNs calculated by *Eisele et al* (PE = 0.49) was combined with the lower uncertainty limit for the proportion of post-neonatal mortality due to malaria [20], [22].

## Results

**Figure 1:**
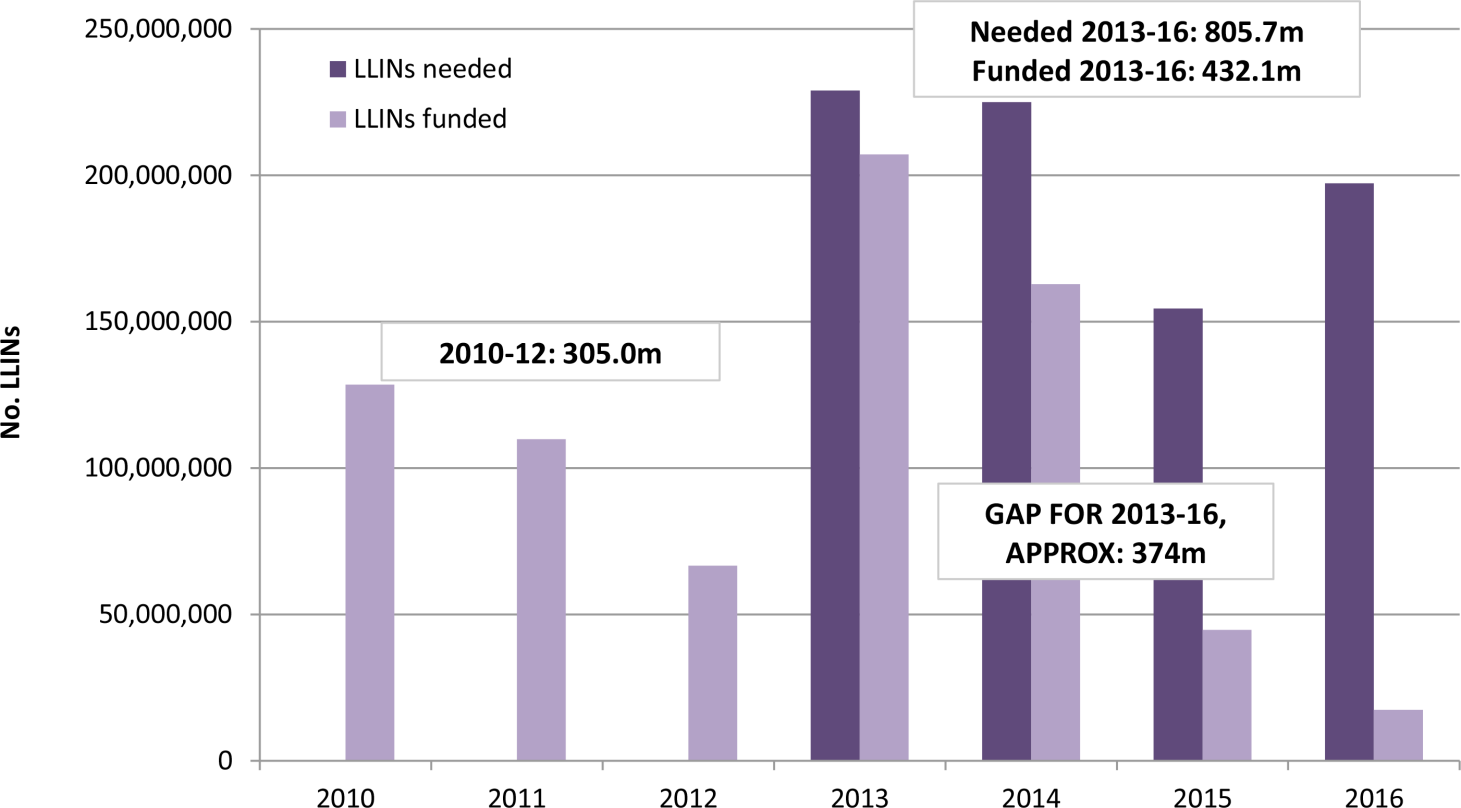
Total LLINs needed and funded for 40 malaria-endemic countries in sub-Saharan Africa (2010–2016).

Between 2010 and 2012, over 300 million LLINs were distributed in 40 sub-Saharan African countries with almost 80 percent of these distributed in 2010 and 2011 (Figure 1). The majority of LLIN distribution in these years was via mass campaigns as part of the considerable efforts by countries to reach the 2010 universal coverage targets. Bottlenecks in grant approvals delayed procurement of LLINs in 2012, contributing to the lower volume of LLINs distributed (M. Renshaw, personal communication).

According to current RBM guidelines, the nets distributed via campaigns in 2010 and 2011 will need replacing in 2013 and 2014, which explains the large LLIN need identified for these years by the country gap analyses (also including making up for the 2012 deficit); the need for 2015 is slightly lower but increases again in 2016, reflecting this three-yearly campaign cycle. Overall, the 40 countries identified a total need of 805.7 million LLINs for the period 2013-16. This is higher than the annual 150 million LLINs identified in the 2012 World Malaria Report, as it includes requirements for all delivery channels rather than the simplified calculation of 1.8 people per LLIN. See Table S1 in File S1) for individual country-level data.

Current funding commitments meet just over half of countries’ needs, leaving a funding gap for approximately 374 million LLINs (Figure 1). Around 90% of LLINs needed for 2013 and 72% of those needed for 2014 are currently funded. However the funding gap is much greater for 2015 and 2016 when only around 30% and 10% of countries’ LLINs requirements have committed funding, respectively. This in part reflects the ongoing replenishment exercises of the Global Fund and World Bank, and also that many of the major donors do not make funding projections this far forward.

**Figure 2:**
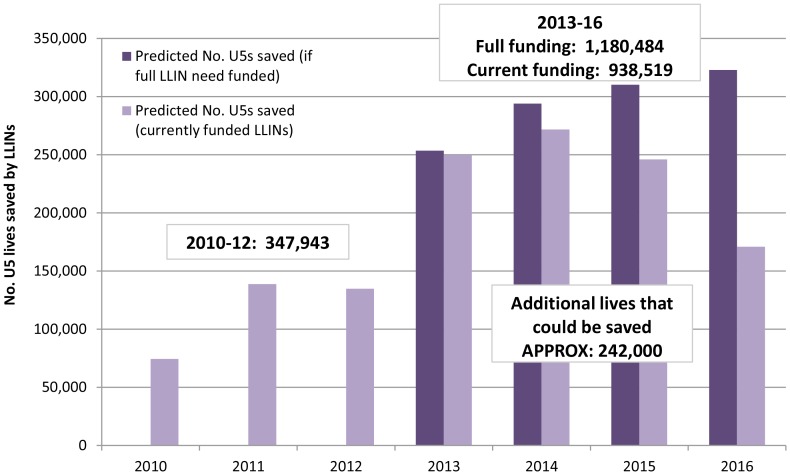
Estimated under-five lives saved with LLINs needed or in the pipeline (2010–2016). Estimated number of under-five lives saved with LLINs distributed between 2010 and 2012, and predictions of the lives that could be saved with full funding for all LLINs needed versus currently funded LLINs in 2013-16.

According to the NetCALC predictions, the LLINs distributed in 2011 and 2012 resulted in a median household ITN ownership of 68% (IQR: 36%, 90%) and 66% (IQR: 34%, 93%), respectively. The median proportion of the population with access to an ITN was 61.6% (IQR: 32.8%, 82.0 %) in 2011 and 60.0% (IQR: 31.1%, 84.6%) in 2012. Although this is a considerable increase from the median household ownership of 33.1% (IQR: 12.0%, 50.8%) and population access to an ITN of 30.0% (IQR: 10.9%, 46.1%) in 2009, only 16 of the 40 countries were predicted to have achieved at least 85% household ITN ownership in at least one year between 2010 and 2012. Based on these annual country-level coverage figures, the LLINs distributed in sub-Saharan Africa between 2010 and 2012 prevented approximately 350,000 additional malaria-attributable under-five deaths compared to the level of LLIN coverage in the ‘baseline’ year of 2009 (Figure 2).

Encouragingly, the LLINs needed and funded for 2013 and 2014 are closer to meeting universal coverage of 100% household ownership and population access to an ITN and have a greater predicted impact on child malaria mortality, with the potential to save an additional 521,600 child lives with current funding and 547,500 if the full identified need was met. The mortality impact gap is greater for 2015 and 2016; if all LLINs needed by countries were funded for these two years then an additional 633,000 child lives could be saved (compared to 2009 LLIN coverage), however with current funding commitments the figure is closer to 416,900, or approximately 65% of the full potential impact.

**Figure 3:**
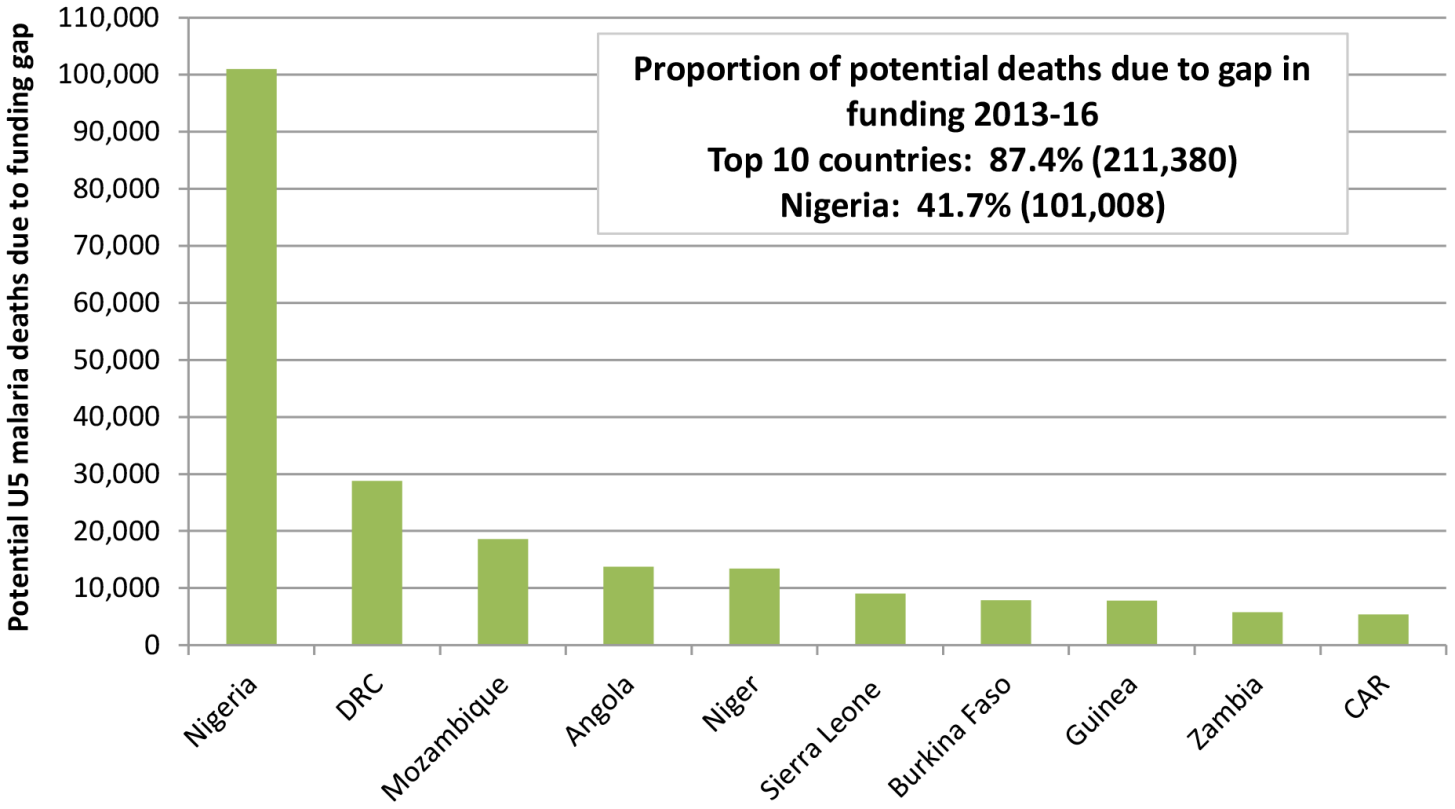
Ten countries with highest mortality gap due to current unmet LLIN funding need. Ten countries with highest mortality gap between what could be achieved if all LLINs were funded versus what will be achieved with current country specific funding for LLINs (CAR = Central African Republic; DRC = Democratic Republic of Congo). These ten countries account for almost 90%, or 211,380 (uncertainty range: 132,061 – 301,810) of these deaths and Nigeria alone accounts for approximately 40%, or 101,008 deaths (uncertainty range: 66,871 – 141,625). Individual country estimates of avoidable deaths (with uncertainty ranges): DRC: 28,000 (14,180 – 42,980); Mozambique: 18,569 (11,594 – 26,260); Angola: 13,779 (7,814 – 20,615); Niger: 13,409 (8,604 – 19,357); Sierra Leone: 9,023 (5,873 – 13,120); Burkina Faso: 7,856 (4,617 – 11,228); Guinea: 7,784 (5,117 – 10,786); Zambia: 5,761 (3,795 – 8,373); CAR: 5,391 (3,596 – 7,466).

Overall, the funding gap for 2013-16 equates to approximately 242,000 avoidable malaria-attributable deaths amongst children under five years over the time period of this analysis (the benefits will however extend beyond 2016). However, almost 90% of these deaths would occur in just 10 of the 40 countries included in the analysis and Nigeria alone accounts for approximately 40% (Figure 3).

Two key assumptions behind the LiST malaria mortality predictions are the proportion of post-neonatal deaths attributable to malaria and the protective efficacy of LLINs. The upper and lower uncertainty limits around these parameters were used to generate a plausible range for predicted mortality impact [20], [22] (Table 1). According to the baseline malaria mortality assumptions applied in LiST, if all LLINs with current funding are distributed between 2013 and 2016 as outlined in national strategic plans then 938,500 child lives could be saved (uncertainty limits: 559,400 – 1,364,200). Conversely, if the additional resources can be mobilised to fully fund the identified need then a further 242,000 child lives could be saved in sub-Saharan Africa over the next four years (uncertainty limits: 147,700 – 354,800) (Table 1).

**Table 1:** Sensitivity analyses of the predicted number of under-five lives that could be saved with full funding for all LLINs needed versus currently funded LLINs, depending on malaria mortality assumptions.

	Predicted no. child lives saved* (lower/upper uncertainty limits)**				
	2013	2014	2015	2016	2013-16
**LLINs required to fully fill estimated needs**	253,500	293,997	310,112	322,875	1,180,484
	(152,220 – 369,022)	(176,162 – 427,878)	(185,456 – 451,565)	(193,198 – 470,478)	(707,036 – 1,718,943)
**Currently funded LLINs**	249,870	271,754	246,010	170,885	938,519
	(150,031 – 363,520)	(162,486 – 395,020)	(145,954 – 357,548)	(100,882 – 248,071)	(559,353 – 1,364,159)
**Mortality gap**	3,630	22,243	64,102	151,990	241,965
	(2,189 – 5,502)	(13,676 – 32,858)	(39,502 – 94,017)	(92,316 – 222,407)	(147,683 – 354,784)

NOTES: *Baseline mortality impact assumptions: PE of LLIN 0.55; default proportion of post-neonatal deaths due to malaria; **Lower mortality impact assumptions: PE of LLIN 0.49; lower uncertainty limit for proportion of post-neonatal deaths due to malaria. Upper mortality impact assumptions: PE of LLIN 0.60; upper uncertainty limit for proportion of post-neonatal deaths due to malaria.

Another important assumption in the analysis is the ITN ownership predicted by NetCALC which is in turn dependent upon the effective lifespan of LLINs. Countries are currently recommended to replace their nets every three years. This figure was therefore used for the NetCALC predictions of household ITN ownership in the present analysis. However, recent data suggest that LLINs may have a shorter average lifespan in certain settings [1]. A reduction in the median lifespan of a cohort of LLINs from three to two years in NetCALC reduced the estimated ITN household ownership that could be achieved annually; in turn this reduced the potential mortality impact. Therefore, rather than a mortality gap of approximately 242,000 avoidable child deaths that could be prevented during 2013-16 if all LLINs were funded and LLINs lasted for three years, the mortality gap increased to 423,500 avoidable child deaths when LLINs were assumed to only last two years.

## Discussion

Country gap analyses have identified that overall a total of 805.7 million LLINs are required to fully meet sub-Saharan African country needs up to 2016. This is higher than the annual 150 million LLINs identified in the 2012 World Malaria Report as it includes requirements for all delivery channels rather than the simplified calculation of dividing the population by 1.8. Current funding commitments are only sufficient to meet just over half of this need (432.1 million LLINs). Around 20 percent of the shortfall applies to 2013 and 2014, with a much larger shortfall anticipated for 2015 and 2016. This pattern is largely due to the expiry of existing Global Fund grants and the short funding cycles of large donors. The shortfall will be reduced as a result of the ongoing replenishment exercises of the Global Fund and World Bank for the period 2014–2016, although the magnitude of commitments is not confirmed at this stage. Additionally it is hoped contributions from other bilateral donors will also increase.

Based on modelling predictions, the scale up of LLINs between 2010 and 2012 is estimated to have saved an additional 348,000 child lives (uncertainty range: 203,200 – 506,700) that would have been lost to malaria, relative to the LLIN coverage in 2009. This is supported by evidence from countries such as Eritrea, Madagascar, Rwanda, Zambia and Zanzibar, which have reported substantial declines in mortality in recent years [1]. With the current funding, these impressive gains will continue with an estimated additional 938,500 child lives (uncertainty range: 559,400 – 1,364,000) that could be saved between 2013 and 2016, relative to LLIN coverage in 2009.

However, much greater impact could be achieved if the full LLIN requirement to achieve and sustain universal coverage were funded. If the resources could be mobilised to fund the current LLIN gap of 373.6 million LLINs for 2013–2016, then an extra 242,000 child lives (uncertainty range: 147,700 – 354,800) could be saved in sub-Saharan Africa, bringing the total impact of LLIN scale-up between 2010 and 2016 to approximately 1.53 million (uncertainty range: 0.91 million – 2.23 million) child lives saved.

It is important to consider these mortality predictions with some caution as there are a number of uncertainties in the parameters used to produce these numbers. However, sensitivity analyses within LiST that incorporated the lower and upper uncertainty limits for proportion of post-neonatal deaths attributable to malaria and the protective efficacy of LLINs go some way to accommodate these uncertainties and so it is likely that the true number of lives that have been saved and could be saved with sustained funding for LLINs lies somewhere in the presented range. Even at the lower end of this range, there are still 707,000 children under five that could be saved over the next four years (compared to LLIN coverage in 2009) if all of the expressed country LLIN needs were funded.

An important assumption in the design of the NetCALC model is that NetCALC predicts the number of LLINs needed annually to maintain a defined coverage level (set here to be 100% for 2013-16) based on population and household size, existing coverage from previous distributions (in this analysis, 2010-12) and median LLIN lifespan. NetCALC calculates the ‘replacement’ need and assumes that all replacement LLINs will reach those not covered in an equitable manner, although does not account for how these LLINs will be distributed; any additional nets planned for distribution above the annual ‘replacement’ level are therefore considered excess by the model. In contrast, during the gap analysis process, countries determine the LLIN need for planned campaigns and continuous distribution delivery channels; this may result in some overlap in recipients and in certain cases a greater LLIN need than the ‘replacement’ need calculated by NetCALC.

This does not mean that the country needs assessments are over-estimated, as it is important to balance minimum net replacement requirements with the operational reality of how LLINs can be delivered to achieve and maintain universal coverage, even if this means some overlap in coverage. The country level data was not disaggregated by proposed delivery channel in the current analysis. However, a recent paper by *Okell et al* confirmed that continuous delivery of LLINs to children at their age of greatest malaria mortality risk provides substantial added benefit in terms of mortality impact compared to campaign delivery alone. Thus some loss of efficiency due to multiple delivery channels is likely to be outweighed by the greater mortality impact [23].

A sensitivity analysis demonstrated the importance of assumptions regarding LLIN lifespan: for example, a reduction in median LLIN lifespan from three years to two years decreased the predicted LLIN coverage and therefore the potential mortality impact. However, LLIN durability appears to vary by site and more data are needed to understand this variation at country level before broader conclusions can be made. Insecticide resistance was not considered, as there has been no operational failure of LLINs observed in the field to date [24] and the time frame of the analysis is only to 2016.

It is important to note that coverage of at-risk populations with IRS was not included in the predictions; hence in certain countries with large-scale IRS programmes (such as Eritrea, Madagascar and Mozambique) some of the mortality gap due to the funding shortfall for LLINs may be averted. Likewise, other malaria interventions such as seasonal malaria chemoprevention or prompt and effective treatment with artemisinin-combination therapy were considered to be static for the purposes of this analysis and may also avert a proportion of the mortality gap if effectively scaled up [25], [26]. Finally, this analysis focuses only on the impact of LLINs on malaria-attributable mortality amongst children under five years, thereby underestimating the full impact of LLINs by not including the child deaths for which malaria is an indirect cause or deaths in other age groups prevented by universal coverage of all individuals at risk [27].

Although the two models used in this analysis, NetCALC and LiST, have some limitations that result in uncertainties with the coverage and mortality estimates, both have been validated by their authors against observed field data [21] (File S2). In addition, the coverage predictions made by NetCALC for the years 2010–2012 based on manufacturer LLIN distribution data were generally close to actual household survey data; the median difference between the two figures (NetCALC prediction *versus* survey data) was 2.3% (IQR: –6.9%, 17.3%) across 23 countries with national survey data available for 2010-12. This provides further validation of the NetCALC model for use across a broad range of country settings and the present analysis.

Over the twenty years since the original ITN trials, many lessons have been learned in the development of distribution strategies to achieve high population coverage with LLINs [28], [29]. Evidence suggests that these efforts and the resources mobilised by domestic and international funders have resulted in impressive achievements in terms of LLIN use [1] and subsequent impact on malaria transmission, morbidity and mortality [1]. However, these gains are fragile [7] and it is imperative that funds and momentum are not lost at this crucial stage. With the current economic crisis, encouraging existing government and multilateral donors to increase their commitments is incredibly challenging and new approaches will likely be needed. The recent Resource Mobilisation Strategy for the 2012-15 phase of implementation of the Global Malaria Action Plan puts forward several suggestions, including increased domestic funding by endemic countries, aid from emerging economies, investment from the private sector and philanthropy, and innovative financing mechanisms such as malaria bonds or air travel levies to meet the shortfall [30]. In addition the new funding model of the Global Fund is intended to improve predictability, in turn improving country planning and encouraging enhanced programming efficiencies. Research and development for better products such as longer lasting nets is also being promoted.

Malaria mortality impact predictions produced by mathematical models are sensitive to levels of uncertainty in key parameters. Nevertheless, this study concludes that without mobilisation of substantial additional resources to meet the full LLIN need of sub-Saharan countries to maintain universal coverage, there is a risk that the impressive gains made to date will be lost and several thousand avoidable child deaths will occur.

## Supporting Information

File S1 **Table S1.** Overview of LLINs needed and currently funded for the period 2013-15 according to country gap analyses, and the number of LLINs distributed in 2010-12 according to comprehensive LLIN manufacturer data.
(DOCX)

File S2 **Comprehensive background document describing the NetCALC model.** This explains the core elements of the model’s underlying calculations and the field data upon which they are based as well as describing the strengths and limitations of the tool.
(PDF)
