# Supplementary material for: Sustaining Fragile Gains: The Need to Maintain Coverage with Long-Lasting Insecticidal Nets for Malaria Control and Likely Implications of Not Doing So
Source: PLoS One. 2013 Dec 27;8(12):e83816. doi: 10.1371/journal.pone.0083816 (PMC3873961; doi:10.1371/journal.pone.0083816)
Supplement: File S1 — Table S1. Overview of LLINs needed and currently funded for the period 2013-15 according to country gap analyses, and the number of LLINs distributed in 2010-12 according to comprehensive LLIN manufacturer data. (DOCX) [file pone.0083816.s001.docx]

Table S1: Overview of LLINs needed and currently funded for the period 2013-15 according to country gap analyses, and the number of LLINs distributed in 2010-12 according to comprehensive LLIN manufacturer data.

|  | **ESTIMATED NEED** | | | | **FUNDED LLIN** | | | | | | |
| --- | --- | --- | --- | --- | --- | --- | --- | --- | --- | --- | --- |
|  | **Country Gap Analyses** | | | | **Net Mapping Project^1^** | | | **Country Gap Analyses** | | | |
| **COUNTRY** | **2013** | **2014** | **2015** | **2016** | **2010** | **2011** | **2012** | **2013** | **2014** | **2015** | **2016** |
| Angola | 3,337,697 | 3,306,022 | 5,241,548 | 3,337,697 | 2,013,059 | 1,212,300 | 2,162,300 | 2,012,500 | 1,900,000 | 1,900,000 | 0 |
| Benin | 689,529 | 6,278,167 | 778,444 | 840,011 | 1,214,345 | 4,311,700 | 207,720 | 689,529 | 6,159,032 | 755,000 | 780,000 |
| Botswana | 60,309 | 187,875 | 62,622 | 63,812 | 76,813 | 0 | 0 | 2,000 | 0 | 0 | 0 |
| Burkina Faso | 10,533,222 | 938,720 | 968,651 | 11,573,971 | 3,041,067 | 2,469,150 | 404,260 | 10,533,223 | 938,720 | 0 | 0 |
| Burundi | 962,824 | 6,129,661 | 992,517 | 880,112 | 3,407,317 | 647,500 | 920,209 | 962,824 | 6,098,979 | 0 | 0 |
| CAR | 2,911,961 | 290,930 | 314,269 | 3,145,585 | 275,000 | 45,750 | 97,720 | 2,055,874 | 133,187 | 137,923 | 0 |
| Cameroon | 1,380,685 | 13,153,843 | 848,612 | 848,612 | 126,343 | 8,918,668 | 358,470 | 361,369 | 13,153,843 | 376,335 | 0 |
| Chad | 2,529,117 | 6,411,464 | 1,041,117 | 1,109,025 | 416,369 | 4,023,415 | 365,354 | 2,529,117 | 6,411,464 | 1,041,117 | 86,400 |
| Comoros | 457,961 | 49,091 | 55,321 | 507,954 | 8,974 | 273,162 | 0 | 445,895 | 20,100 | 25,800 | 0 |
| Congo | 133,539 | 2,084,856 | 573,900 | 573,900 | 1,138,301 | 1,759,903 | 152,960 | 133,539 | 0 | 0 | 0 |
| Cote d'Ivoire | 1,419,064 | 15,973,302 | 1,940,043 | 2,137,668 | 6,036,823 | 2,443,100 | 1,015,900 | 1,419,064 | 13,911,841 | 500,000 | 500,000 |
| Djibouti | 600,121 | 214,000 | 455,121 | 0 | 28,500 | 20,200 | 26,400 | 455,121 | 0 | 0 | 0 |
| DRC | 15,814,897 | 27,937,150 | 16,332,757 | 16,417,893 | 13,048,638 | 17,785,593 | 1,749,877 | 15,814,897 | 19,701,028 | 0 | 250,000 |
| Eritrea | 1,257,703 | 88,592 | 211,809 | 1,257,703 | 233,750 | 1,266,200 | 117,500 | 1,257,703 | 88,592 | 0 | 0 |
| Ethiopia | 24,383,164 | 7,039,514 | 12,163,720 | 19,398,836 | 12,191,978 | 3,576,300 | 6,523,303 | 16,995,759 | 4,986,332 | 0 | 0 |
| Gabon | 78,330 | 1,265,456 | 108,672 | 149,240 | 121,270 | 19,800 | 40,818 | 21,820 | 8,900 | 9,022 | 9,200 |
| Gambia | 116,795 | 1,114,832 | 122,334 | 122,334 | 247,278 | 585,596 | 348,825 | 116,795 | 965,849 | 122,334 | 0 |
| Ghana | 2,885,380 | 16,576,652 | 2,388,714 | 1,778,863 | 2,258,757 | 6,857,289 | 7,019,344 | 1,820,000 | 9,556,333 | 2,388,714 | 1,020,000 |
| Guinea | 7,182,987 | 894,367 | 949,391 | 7,984,507 | 877,820 | 38,187 | 116,100 | 6,011,222 | 0 | 0 | 0 |
| Guinea Bissau | 134,285 | 1,087,061 | 147,207 | 150,225 | 20,027 | 903,439 | 279,000 | 134,285 | 830,790 | 15,869 | 0 |
| Kenya | 1,732,116 | 14,520,577 | 1,934,318 | 1,992,347 | 5,369,047 | 11,905,798 | 5,760,312 | 1,732,116 | 10,416,359 | 1,750,000 | 0 |
| Liberia | 1,093,990 | 976,039 | 1,586,183 | 1,182,113 | 809,591 | 1,170,000 | 1,103,415 | 657,327 | 909,089 | 276,427 | 0 |
| Madagascar | 6,996,408 | 1,800,000 | 11,181,374 | 2,300,000 | 5,060,425 | 40,560 | 3,351,000 | 6,735,320 | 1,800,000 | 3,001,053 | 2,300,000 |
| Malawi | 4,249,679 | 1,580,524 | 10,692,393 | 1,683,291 | 1,367,591 | 1,183,800 | 6,946,585 | 4,037,311 | 900,000 | 7,900,000 | 900,000 |
| Mali | 1,505,094 | 1,559,278 | 11,587,088 | 1,673,566 | 1,097,547 | 3,827,800 | 1,190,650 | 1,505,094 | 1,559,278 | 4,097,349 | 600,000 |
| Mauritania | 1,493,774 | 178,922 | 193,278 | 1,626,996 | 100,000 | 52,200 | 0 | 0 | 0 | 0 | 0 |
| Mozambique | 4,826,433 | 7,494,962 | 5,377,759 | 7,032,821 | 956,382 | 3,721,046 | 4,633,423 | 4,826,433 | 1,465,839 | 0 | 0 |
| Niger | 6,126,745 | 1,431,422 | 1,594,193 | 6,992,223 | 775,225 | 428,350 | 328,662 | 6,080,664 | 444,689 | 439,250 | 439,250 |
| Nigeria | 62,586,372 | 33,591,811 | 42,346,674 | 33,788,224 | 34,139,729 | 3,247,976 | 3,648,314 | 62,586,372 | 17,383,215 | 5,031,711 | 1,556,711 |
| Rwanda | 7,253,198 | 1,429,344 | 1,385,304 | 8,483,284 | 4,852,939 | 1,442,294 | 2,710,111 | 5,968,925 | 1,429,344 | 0 | 0 |
| Senegal | 4,340,000 | 3,779,525 | 2,500,000 | 942,398 | 2,281,905 | 2,929,950 | 45,000 | 4,340,000 | 3,779,525 | 2,500,000 | 0 |
| Sierra Leone | 3,911,327 | 497,923 | 539,628 | 4,268,717 | 3,353,818 | 141,300 | 799,268 | 796,384 | 497,923 | 535,031 | 548,407 |
| Somalia | 575,000 | 615,000 | 1,625,000 | 575,000 | 75,000 | 238,000 | 401,104 | 575,000 | 615,000 | 1,625,000 | 0 |
| Sudan^2^ | 15,503,593 | 4,308,237 | 5,225,099 | 14,332,610 | 3,678,546 | 367,150 | 6,261,336 | 14,667,785 | 2,890,416 | 5,225,099 | 6,769,177 |
| Swaziland | 21,864 | 103,582 | 49,066 | 26,446 | 114,656 | 0 | 0 | 21,864 | 103,582 | 49,066 | 8,938 |
| Tanzania^3^ | 3,771,174 | 25,405,123 | 4,579,245 | 10,436,304 | 5,582,710 | 14,514,490 | 1,969,520 | 3,766,945 | 25,348,726 | 712,222 | 0 |
| Togo | 884,611 | 4,243,533 | 412,649 | 429,597 | 251,482 | 2,769,854 | 20,000 | 827,795 | 3,694,863 | 412,649 | 74,797 |
| Uganda | 22,182,147 | 2,786,110 | 3,035,526 | 24,839,701 | 8,610,413 | 880,747 | 1,636,800 | 22,182,147 | 2,622,954 | 2,422,355 | 550,000 |
| Zambia | 1,443,227 | 5,632,573 | 1,156,288 | 1,190,976 | 1,617,605 | 3,704,014 | 3,178,930 | 1,443,227 | 1,146,433 | 840,000 | 440,000 |
| Zimbabwe | 1,536,099 | 1,552,996 | 1,570,079 | 1,073,034 | 1,411,432 | 0 | 480,307 | 500,000 | 500,000 | 500,000 | 500,000 |
| **TOTAL** | **228,980,998** | **225,017,727** | **154,462,984** | **197,231,154** | **128,506,068** | **113,690,144** | **66,632,563** | **207,091,419** | **162,870,077** | **44,761,256** | **17,404,990** |

*NOTES: ^1^ A lag time of 3 months has been applied to the Net Mapping Project data as a proxy for the time between LLINs arriving in country and distribution to recipients; ^2^ Sudan and South Sudan considered together for the purposes of this analysis as data not currently available separately in LiST for mortality predictions; ^3^Data for Tanzania includes Zanzibar as data not currently available separately in LiST.*

*ABBREVIATIONS: CAR = Central African Republic; DRC = Democratic Republic of Congo; STP = Sao Tome & Principe.*
