# Supplementary material for: Sustaining Fragile Gains: The Need to Maintain Coverage with Long-Lasting Insecticidal Nets for Malaria Control and Likely Implications of Not Doing So
Source: PLoS One. 2013 Dec 27;8(12):e83816. doi: 10.1371/journal.pone.0083816 (PMC3873961; doi:10.1371/journal.pone.0083816)
Supplement: File S2 — Comprehensive background document describing the NetCALC model. This explains the core elements of the model’s underlying calculations and the field data upon which they are based as well as describing the strengths and limitations of the tool. (PDF) [file pone.0083816.s002.pdf]

NetCALC 2.0 Background Information

Table of Content

History and Development 2
Basic design 2
Loss function........ 3
Coverage estimation 7
Adjustments and additions 10
Net treatment and re-treatment 13
Inputs needed........ 14
Limitations........ 15

Introduction

This objective of this document is to:

- introduce users of NetCALC to the background and history of the tool
- explain the core elements of the underlying calculations and how they were arrived at
- explain the strengths and limitations of the tool

History and Development

The precursor of what is now NetCALC was created in 2005, by Dr. Albert Kilian, as a simple 3-page spreadsheet during his work at the National Malaria Control Program (NMCP) in Uganda. In writing a Global Fund proposal, the NMCP Uganda were challenged by the question "how many nets do we need to reach the national target for 80% household coverage?" and it was his task to undertake the necessary estimations. NetCALC essentially had only one function that was based on the 2001 census data and population growth in Uganda from 2005-2012 and used the resulting number of households as well as the nets distributed to date to calculate resulting net ownership coverage. From there it has grown consistently over time and is now applicable beyond Uganda as a very valuable tool for program managers to assist them in the planning and management of malaria prevention with insecticide treated nets (ITN).

In 2011, Dr. Kilian worked on the NetWorks[1] project through one of the project partners, Malaria Consortium, and together they increasingly saw the need for a tool to predict long-lasting insecticidal net (LLIN) needs during campaigns and continuous distribution programs. The original tool was abandoned and re-written with the main objective of making it user friendly. Microsoft Excel was chosen as the software because most people are familiar with it and since Dr. Kilian felt comfortable using Excel to construct the necessary functions. In the process more and more modules were added that had not existed in the previous version. For example, NetCALC initially only allowed for antenatal care (ANC) distributions of nets, but in 2011 modules for distribution via curative health services, schools or communities or the retail market. In 2012, for version 2.0, the "proportion of population with access to ITN within the household" was added as a new output variable.

Basic Design

The principal design of the NetCALC tool is a simple, compartmental model, i.e. a model where quantities move from one compartment (or category) to the next at a defined rate. It is based on populations rather than individual people or households and has two major components. The first component that needs to be estimated is called the **net crop** or the number of nets available in a given system at a given time. Example: in country A at in the end of year X, how many nets are available in the households to be used by the population? The net crop essentially depends on what is going in (i.e., the number of nets distributed or sold in a year) and on what is going out, or the loss function (i.e., the number of nets that were lost due to wear and tear or using the nets for other purposes than sleeping under). The second component translates this net crop into an outcome measurement or **coverage rates**. By making some assumptions on how nets accumulate within the household as their availability increases and their distribution within the household, this component calculates two of the currently recommended Roll Back Malaria (RBM) indicators: "proportion of households with at least one ITN" and "proportion of the population with access to an ITN within the household."

Since these two components are the core of NetCALC, below is a more detailed description of the creation of the various formulas.

---

[1] http://www.networksmalaria.org/

Figure 1: The principle design of the compartmental model

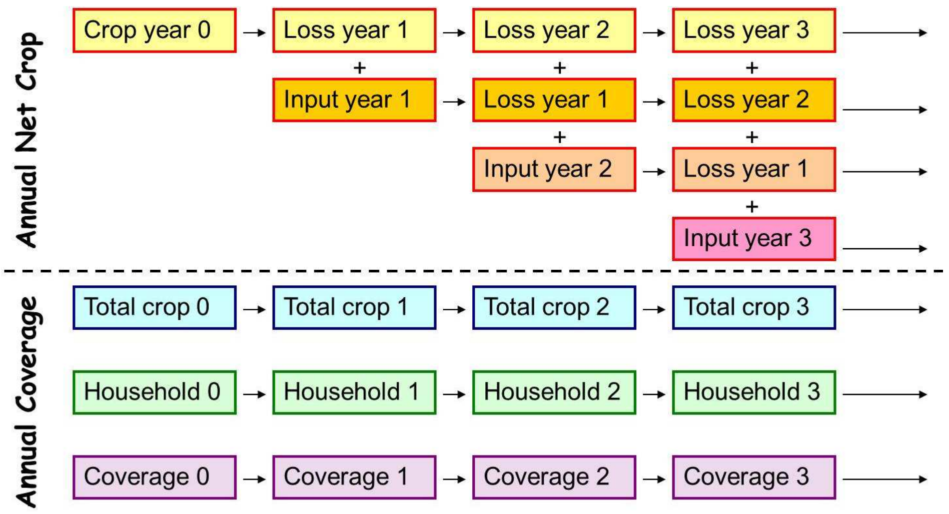

Loss function

When work began on this type of module in 2004/05, no one was thinking or talking about net loss or net durability. However, some information about it existed from cross-sectional surveys. For example, information existed on estimates of the proportion of households with nets and the number of nets in each of these households (i.e. the mean number of nets). This data could be used to calculate how many nets were in that population at different points in time. Information was also available on the age of the nets found in surveys so that an age-distribution curve could be constructed giving some indication about net survival. Later, follow-up surveys after campaign distributions were added which also allowed an estimation of the net loss in the first months or years following distribution.

For example, data from the Uganda Demographic and Health Survey (DHS) in 2001, as well as the HIV/AIDS Survey in 2005 was found in which the following two net related questions were asked of respondents: (1) Do you have any mosquito nets and (2) how many do you have? With this data, it was possible to calculate the estimated net crop at the national level for each time point. This was supplemented by the number of nets sold or distributed from all sources (public and commercial) in the time between the two surveys so that the initial crop plus net input could be compared to the crop at the second survey; the difference between these would reflect the net loss during the time period. Using a range of estimates for the informal commercial sector, the results in Table 1 below show the magnitude of the loss within the four years.

Table 1: Estimated net loss

	Low estimate	High estimate
Total nets	3,299,020	4,001,020
Loss estimate	734,713	1,436,713
% loss	22.2%	35.9%

During most household surveys such as DHS or the Malaria Indicator Survey (MIS), the age of the nets (how long ago did you obtain this net?) is only recorded up to 36 months and then grouped as "3 years or older," making an analysis of the age-distribution of existing nets impossible. However, in

Uganda a number of regional or district surveys were carried out recording the full age of all nets in months or years. An age distribution curve (kernel density) from 12 surveys in Uganda (> 2500 nets) is shown in Figure 2. While the initial increasing part is difficult to interpret with respect to loss, as the peak or mean will depend very much on the level of net distributions, there is a very long tail indicating that some nets survive for quite some time. You can see the fraction of nets older than three year is very small (~1-2% or less) but it is important to note that that the tail does exist.
Dr. Kilian, who was collecting nets every six months from the World Health Organization's Pesticide Evaluation Scheme (WHOPES) phase III LLIN studies, discovered that a number of nets were torn and ready to be thrown away after only 6-12 months. It was also clear that there must be very early loss of nets that, though small, does exist; campaigns do not wait two years before net loss begins. Therefore, any loss function would have to have moderate initial loss, a steep middle and a long tail.

Figure 2: Age distribution of nets from 12 household surveys in Uganda (N=2569)

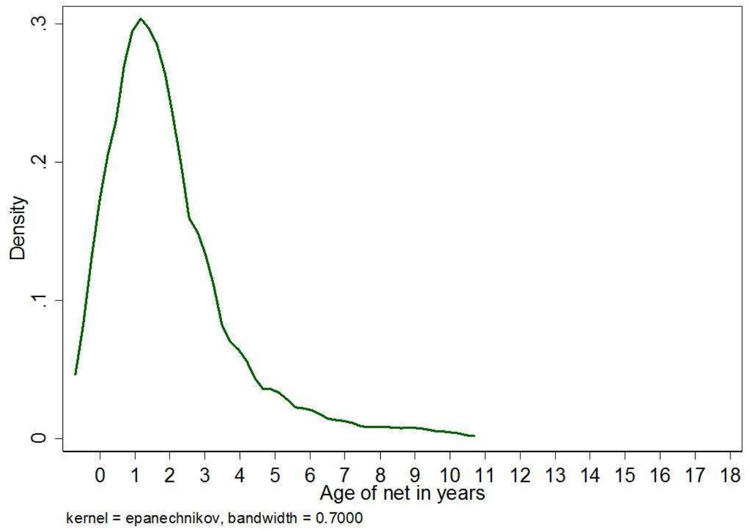

Based on his information an S-shaped curve was manually constructed a where the loss function would have a median survival of either three or five years, reflecting thinking on net survival at the time.[2] This appeared to be a better representation of the available information compared to a fixed loss rate, say 20%, which would over-estimate losses in the beginning and under-estimate losses at the middle section (Figure 3). However, it was obvious for a generally applicable tool that NetCALC needed a more professionally constructed loss function.

Dr. Kilian presented his manual attempts to some colleagues, and it was Nakul Chitnis from the Swiss Tropical and Public Health Institute in Basel who created a formula for the NetCALC loss function, shown in Box 1. It has two parameters, *k* and *L*, in addition to the time variable, and with that it produces an S-shaped curve that can be modulated by changing the values for *k* and *L* producing curves with defined medium survival.

[2] Current thinking was that 150 Denier polyethylene nets would have a survival of around five years and 75 Denier polyester nets around three years (this hypothesis could since not really be confirmed).

Figure 3: Initial attempts to construct a loss function based on available information compared to a fixed rate function (20% loss)

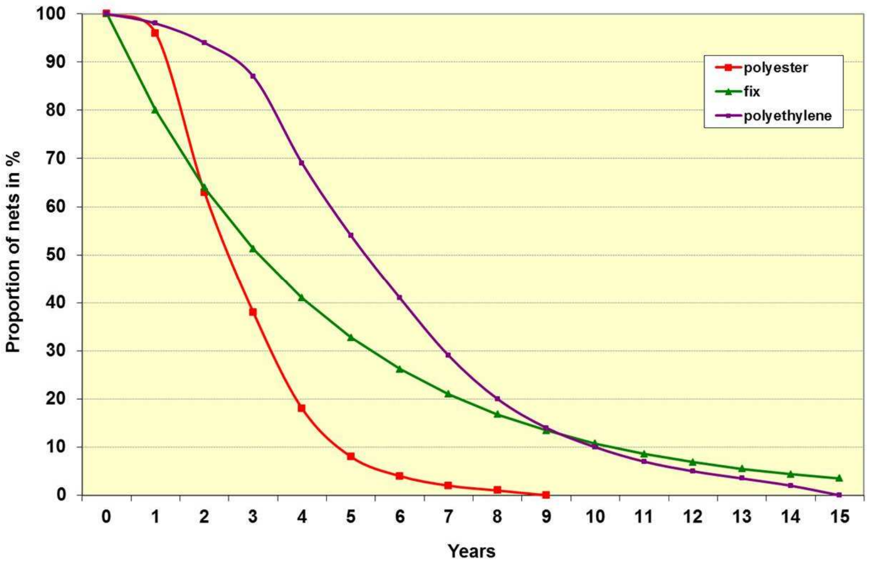

Box 1: Loss function

$$for\ t < L: \quad f(t) = 100 \times \exp\left[k - \frac{k}{(1 - t/L)^2}\right]$$

$$for\ t \geq L: \quad f(t) = 0$$

Where
t=years since distribution of net

The question remained: how do these loss functions compare to actual data from the field? Figure 4 presents the three and five year curves from NetCALC and plots them against all currently available data from published and unpublished surveys (i.e. from retention surveys, post-campaign surveys, and unpublished data from Malaria Consortium). As you can see, there is a lot of data from the first year or so, which is a result of all of the post-campaign surveys with retention rates somewhere between 90-98%. The losses here are not due to wear and tear but rather to redistribution of nets outside of the household due to over-supplied by the campaign. The red dots are polyester 75 Denier nets; the blue dots are polyethylene 150 Denier nets; and the green dots are indirect data. You see that there is one point at five years which represents a survey that was recently published reporting 11.7% of originally distributed nets still present. If you put these all together, they actually seem to roll quite nicely in between the 3-5 year bracket. However, there also exist some outliers (not shown in Figure 4) which do not represent the "real life" situation since they either come from results of longitudinal studies (e.g. WHOPES phase III) where people know that the nets will be followed up and do not throw them away irrespective of their condition or community trials.

Figure 4: Comparison of three and five-year loss curves from model (lines) with data from retention surveys (red squares 75D polyester nets; blue squares 150D polyethylene nets) and indirect data comparing household ownership rates drop from consecutive surveys (green squares).

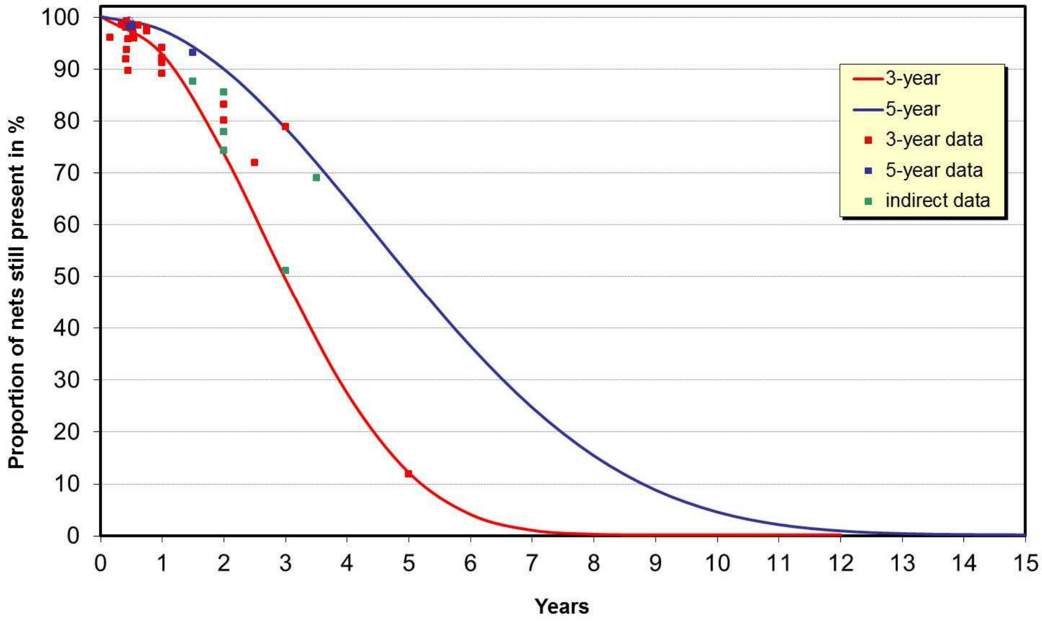

Based on this, using these loss functions from 2-7 years in NetCALC appeared to be a reasonable approach. If the existing data proves to be totally wrong, which is doubtful, and the curve should look different, the loss function can be changed easy enough. This is what is now installed in NetCALC where you can choose what survival rate you want to use. The functions you can select in NetCALC are shown in Figure 5 with the corresponding *k* and *L* values given in Table 2.

Figure 5: The loss functions installed in NetCALC

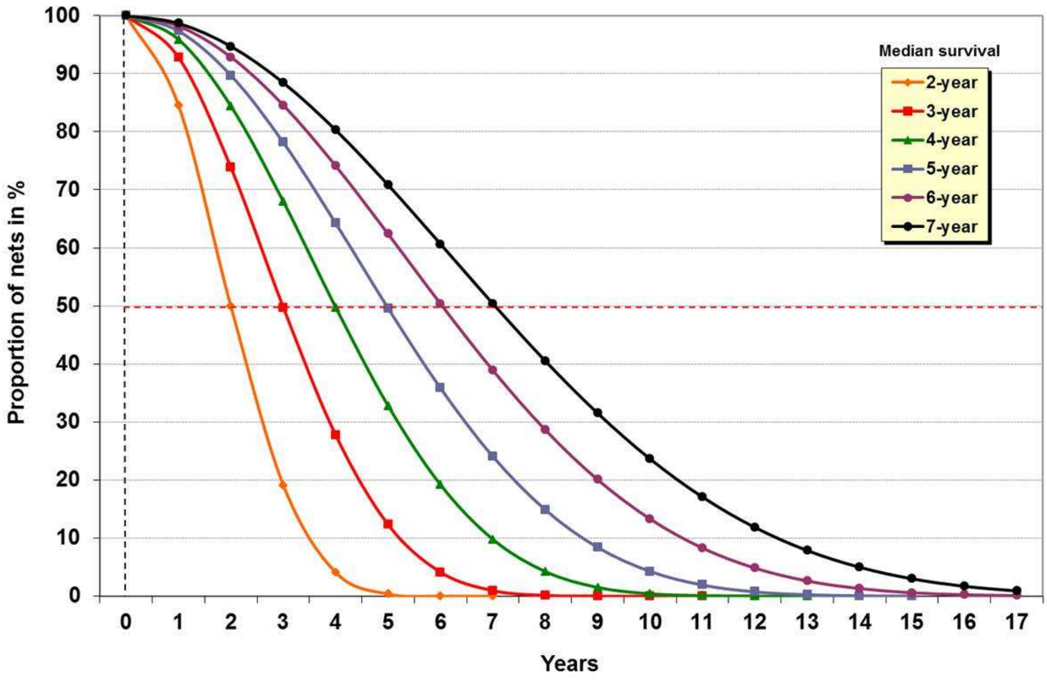

Table 2: Values of k and L used in the loss functions

Median net survival in years	k	L
2	16	9.8
3	17	15.1
4	18	20.7
5	19	26.5
6	22	34.5
7	24	42.0

Coverage estimation

With the loss function established we now come to the second component: how does one translate a net crop into a coverage rate? At first, only the “proportion of households with at least one net or ITN” was considered as it was at the time the only indicator in use.
Starting point was data from a number of surveys from Uganda at a time when no mass campaign had yet taken place and almost all nets were from the commercial sector or social marketing. Plotting the coverage rate (at least one net per household) in a given survey or district, disaggregated by rural and urban against the mean number of nets per net owning household (meaning if you have any nets, how many do you have) showed that these two variables were reasonably well correlated (Figure 6). Essentially, when coverage increases, the accumulation of nets within houses also increases. Moreover, data from surveys from Zambia, Sudan and Malawi strongly suggested that the phenomenon was not limited to Uganda, with data showing the same gradient in the regression analysis although the constant changed according to household size. Therefore, this association could be used to predict the coverage rate if the “mean number of nets per net owning household” was known.

Figure 6: Data from Ugandan household surveys showing correlation between ownership coverage and mean number of nets per net owning household (solid circles=rural; open circles=urban)

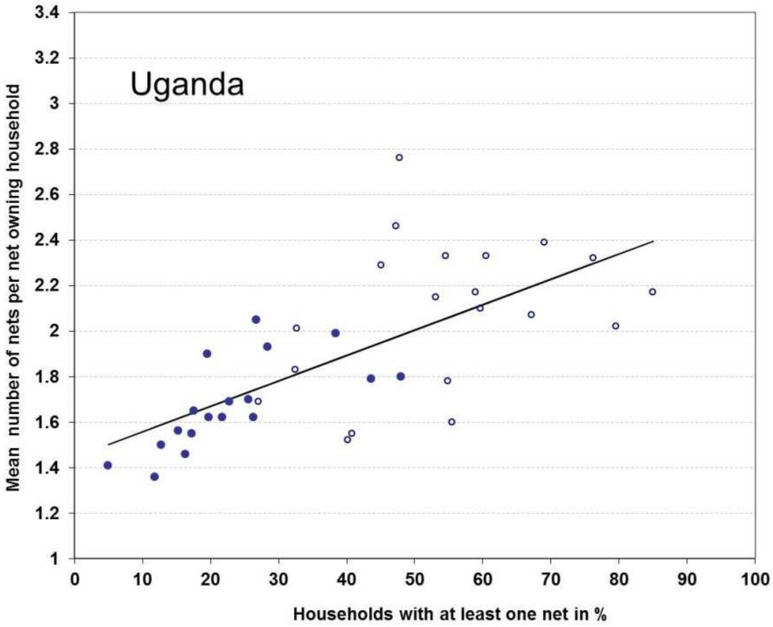

However, the model only had the total number of nets at time x and the number of households in the population at that time. So an additional step was needed to define a relationship between the mean number of nets per net owning household and the mean number of nets per all households, i.e. the ratio between nets and households, which is in the model. This was done by creating an example of the range of coverage rates and corresponding mean number of nets per net owning households from the previous linear regression and calculating the corresponding ratio of nets to households. Plotting the results showed a curvilinear function (Figure 7, below left).

Subsequently, the relationship was modeled in a statistics program (STATA) using a fractional polynomial regression (the fracpoly command). This means that STATA looks through a number of different models and identifies the one model that has an optimum between complexity and simplicity for the relationship. It found a model with two terms and an R-squared value of 99.9% which means the model can almost exactly predict the data (Figure 7, below right). Details of the polynomial function are given in Box 2.

At this point, NetCALC could calculate the "mean number of nets per net owning household" from the number of nets and the households in the model, and with that was able to use the original regression analysis to estimate the "proportion of households with at least one net/ITN". It is important to understand that "at least" one net implies that the higher the coverage the more nets there will be within the household and at 100% there would be "enough nets for everyone in the household".

Figure 7: Relationship between "mean number of nets per net owning household" and "mean number of nets per household" as a function of ownership coverage (left) and the polynomial function translating mean nets per household into mean per net owning household.

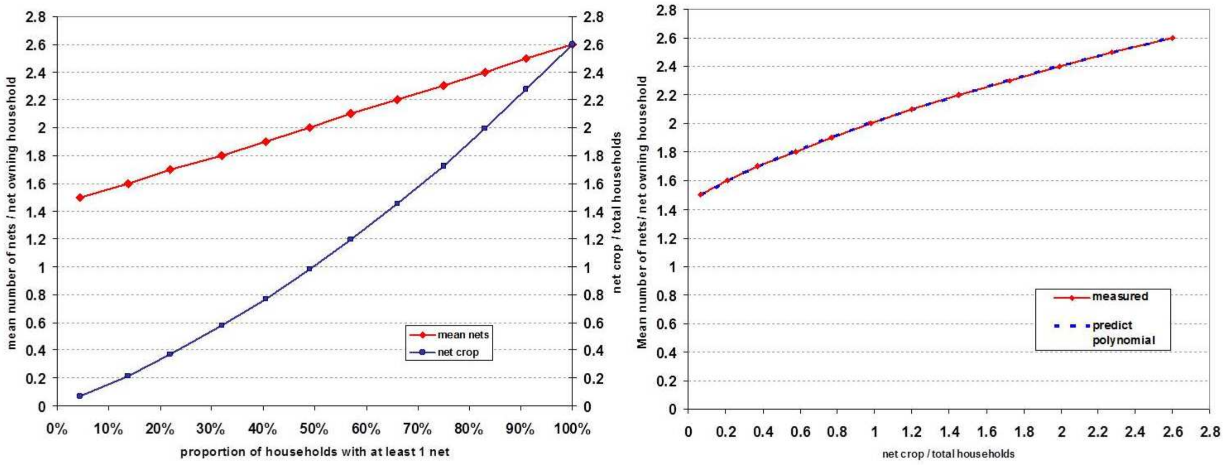

The critical questions that remain are: can the model be validated with real data? Does the model accurately predict the net ownership coverage?

Data from Uganda is shown in Figure 8. Based on the 2001 DHS, you can see only 14% of households had any net and 1.6% had any ITN, therefore the model was calibrated with these numbers as the starting point. Data from the number of nets that were distributed per year in Uganda after 2001, including from the retail market (orange bars) was then entered into the model assuming a three-year loss function with the resulting output for any net ownership (blue line) and any ITN ownership (red line). Data points from subsequent surveys in Uganda, the 2006 DHS and 2009 MIS were then added and, as can be seen in Figure 8, the predictions are reasonably close (within 3-4% points). With these results, it was determined that NetCALC could predict the coverage rate found in the surveys

from only inputting the number of nets per year, i.e. that, at least in Uganda, the model worked well to predict what was confirmed by surveys later.

Figure 8: Estimated coverage with any net (blue line) and any ITN (red line) from NetCALC based on net input and results from the 2006 Demographic and Health Survey (blue diamonds) and the 2009 Malaria Indicator survey (purple diamonds) in Uganda.

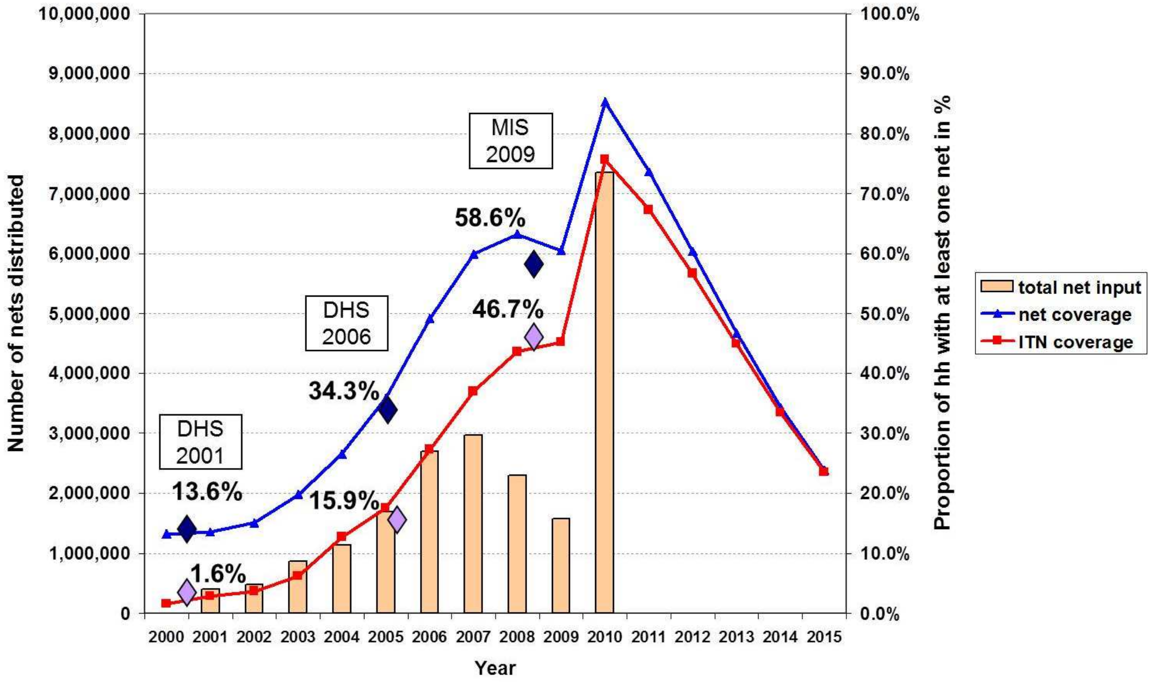

The question remained: would the model work for countries other than Uganda? In Mozambique data were available from two provinces, Sofala and Manica, where a mass distribution had been carried out in 2004 with a follow-up survey done by the Centers for Disease Control (CDC) in 2005 and the MIS in 2007 with provincial results. Data from the 2003 DHS was used with very low net numbers to calibrate the model. After, the total number of nets distributed per year since 2003, data that were available from the National Malaria Control Program (NMCP) and partners in Mozambique (with help from Tim Freeman, UNICEF), was entered into the model. Figure 9 shows the results for Sofala Province but the Manica data is very similar. The CDC survey found a net ownership coverage of 59% after the campaign: the predicted 60%. For ITNs, the CDC survey found 48%; the model predicted 45%. Similarly, for the 2007 MIS survey, the model predicted 50% coverage and the survey found 51%; for ITNs, the model predicted 26% and the MIS survey found 22%. Therefore, even in a setting outside Uganda (but with similar mean household size), the model can predict the coverage rate with reasonable accuracy.

Nonetheless, there are places where the model does not work, specifically if the mean household size is either much larger (e.g. in Senegal) or much smaller and it was clear that NetCALC needed a more exact adjustment for the actual household size to be generally applicable.

Figure 9: Estimated coverage with any net (blue line) and any ITN (red line) based on net inputs in NetCALC with data from household surveys in Sofala Province, Mozambique.

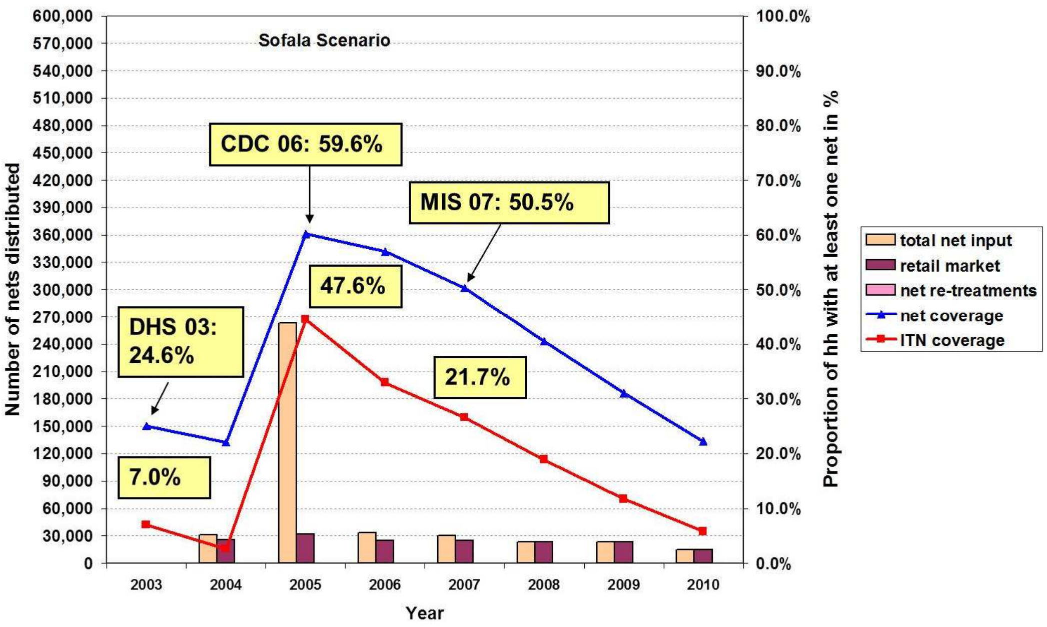

Adjustments and additions

Adjustment for household size

It was clear that the variable "mean number of nets per net owning household" in the model had to be adjusted to account for "number of people in household" in order to make it generally applicable to any country.

In addition, a 2011 recommendation issued by WHO and RBM stated that the quantification of the number of ITN needed to achieve "universal coverage", i.e. one net for every two people in each household, should be done by dividing the population by 1.8 rather than 2.0, as was initially suggested, in order to account for odd-numbered households. If NetCALC was to be used as a tool for national programs to quantify their net needs, it had to ensure that the "universal coverage" in the model reflected this calculation algorithm.

As mentioned earlier, the formula used in the model derived from the Uganda data already takes into account full intra-household saturation with nets at 100% coverage. Interestingly, with an average household size of 5.0 in Uganda, the 2.6 nets per household at 100% coverage are equivalent to a factor of the population divided by 1.9, which is very close to the WHO/RBM recommendation of 1.8.

Implementation of the two new adjustments in NetCALC:

With a hypothetical population of 2 million people, the number of nets at 100% coverage was calculated from the original model. The household size was then varied from 2.0 to 10.0 and the correction factor to get to 100% coverage with that household size was manually determined. Lastly, the difference between the "mean nets per net owning household" from the model and the corrected number was plotted (Figure 10).

This turned out to be a linear function and therefore it was straightforward to run a regression analysis. This analysis predicts the correct "mean number of net per net owning household" adequate for the mean household size of a given population to be used for the net ownership coverage estimation. This, at 100% coverage, would be equivalent to the population divided by 1.8.

Box 2 gives the exact formula and coefficients used in NetCALC. It takes the original calculation, what was derived for Uganda, and adds the correction factor. While this may sound a bit like patch work, it was easier than totally reconstructing the relationship of nets perhousehold to coverage.

Figure 10: Correction of "mean nets per net owning household"from Uganda data to adjust for household size and WHO algorithm for universal coverage (population/1.8). First for each household size the mean was calculated with the Uganda formula and then manually corrected to reach 100% coverage (left) the difference between the means was then plotted against household size and the regression used as the correction function (right).

Box 2: Ownership coverage (c_o) of households with at least one net (ITN, LLIN)

$$c_o = \frac{m}{s_{uc}}$$

with

$$\mathrm{m} = \frac{n}{h}$$

$$s_{uc} = -0.01254 + 0.55431(r) + 0.00717(s_x)$$

$$s_x = 2.088902 + 0.5732837(\mathrm{m} - 1.185) - 0.1299288(\mathrm{m} \times \ln(m) - 0.2011)$$

where

s_x= mean number of nets per net owning households from Uganda data
s_{uc}=mean number of nets per net owning households adjusted to universal coverage
h= number of households in population
m=mean number of nets (ITN, LLIN) per household in population
n= number of nets (ITN, LLIN) in population
r= mean number of persons per household

hh	213,220
net crop	400,000
mean net	2.36
coverage	79.6%
pop	2,000,000
pers/hh	3.50
hh	571,429
netcrop	1,111,111
mean net/nethh	2.38
corrected for pers/hh	1.945
coverage	100.0%

pers/hh	mean net/nethh	100	diff	predicted
2.00	2.06	1.111	-0.949	1.111
3.00	2.28	1.666	-0.614	1.667
3.25	2.33	1.805	-0.525	1.806
3.50	2.38	1.945	-0.435	1.945
3.75	2.43	2.084	-0.346	2.084
4.00	2.48	2.223	-0.257	2.222
4.25	2.53	2.360	-0.170	2.361
4.50	2.57	2.500	-0.070	2.500
4.69	2.61	2.606	-0.004	2.606
4.64	2.60	2.580	-0.020	2.578
4.70	2.61	2.612	0.002	2.611
4.80	2.62	2.667	0.047	2.667
4.90	2.64	2.722	0.082	2.723
5.00	2.66	2.779	0.119	2.778
5.10	2.68	2.834	0.154	2.834
5.25	2.70	2.916	0.216	2.917
5.50	2.74	3.055	0.315	3.056
5.75	2.78	3.195	0.415	3.195
6.00	2.83	3.334	0.504	3.334
6.25	2.86	3.472	0.612	3.472
6.50	2.90	3.611	0.711	3.611
6.75	2.94	3.750	0.810	3.750
7.00	2.98	3.888	0.908	3.889
8.00	3.12	4.444	1.324	4.444
9.00	3.26	5.000	1.740	5.000
10.00	3.38	5.555	2.175	5.555

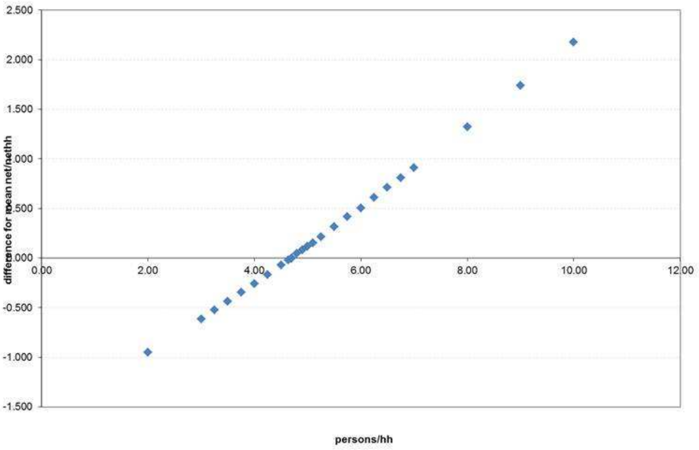

<u>Addition of access coverage rate</u>
In the context of developing a new set of indicators to assess "universal coverage" with ITN, the RBM Monitoring & Evaluation Reference Group (MERG) developed some additional indicators that are now recommended by WHO. One of these indicators is "access of the population to an ITN within the household," assuming that each net is used by two people on average (which we know from many surveys is a very reasonable assumption). This gives us an idea of what proportion of people could use a net or ITN, and comparing it to the proportion actually using a net or ITN defines the "use gap" in contrast to the "ownership gap."
Obviously a model like NetCALC cannot estimate a behavioral aspect such as net use since it only has the number of nets and the population and households to work with. But access is defined by these two variables and adding it to NetCALC seemed an important improvement of the tool.

However, just using the formula ITN*2/population will not give us the desired result because of the "odd numbered household" problem (Figure 11):
Let's assume we have 2 households. One has 2 LLIN and 5 people and the other has 4 LLIN and 7 people, so together we have 12 people and 6 nets and according to the formula LLIN*2/population 100% or the population has access. But there are 2 nets in the first house so only 4 people can sleep under a net and one cannot. And if there are 4 nets in the other house, all the 7 people can sleep under a net and there is space left for one more person. However, the one person without access to a net cannot use the free net space because net and person are not in the same house. Therefore, we need some correction factor to estimate our access indicator.

Figure 11: A simple example to demonstrate why "ITN*2/population" will always over-estimate access coverage due to mismatches in location between people and nets. Box A shows the calculations and Box B the true coverage.

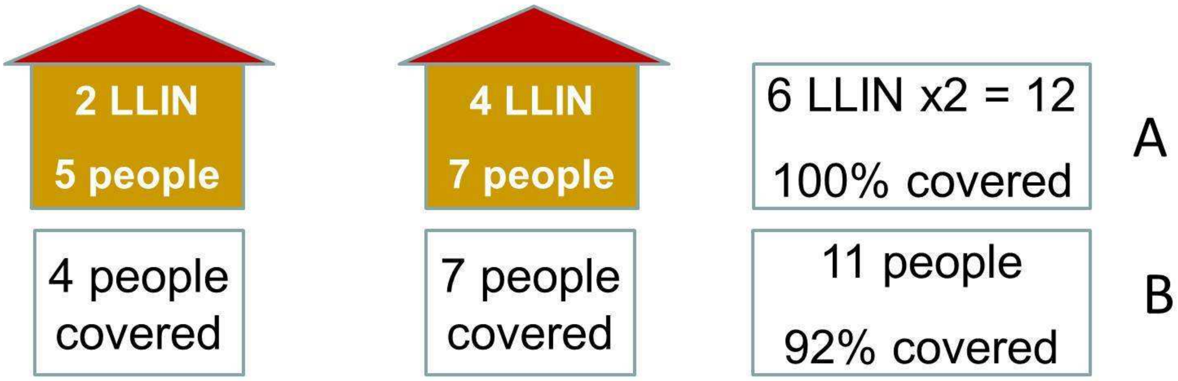

In order to determine the correction factor 17 data sets from household surveys with a large range of "access" outcomes were used and the access variable was plotted against the ratio ITN/population (Figure 12). It shows that there is a reasonable linear correlation so that a linear regression is the best solution. In order to ensure that nobody has access to a net when there are no nets in the system at all the regression analysis was run with a "no constant" option so that the regression line was forced to go through zero. As a result, the optimal correction factor is 1.64 (exact formula see Box 3). The green triangles in Figure 12 show the predicted values from the nets and people, which are nicely

around the equity line, against the actual access indicator from the survey. With this correction factor NetCALC can now also estimate the “proportion of population with access to ITN”.

Figure 12: Relationship between the ITN/100 people and the access coverage rate from 17 household surveys (red circles) used to predict the access coverage (green triangles).

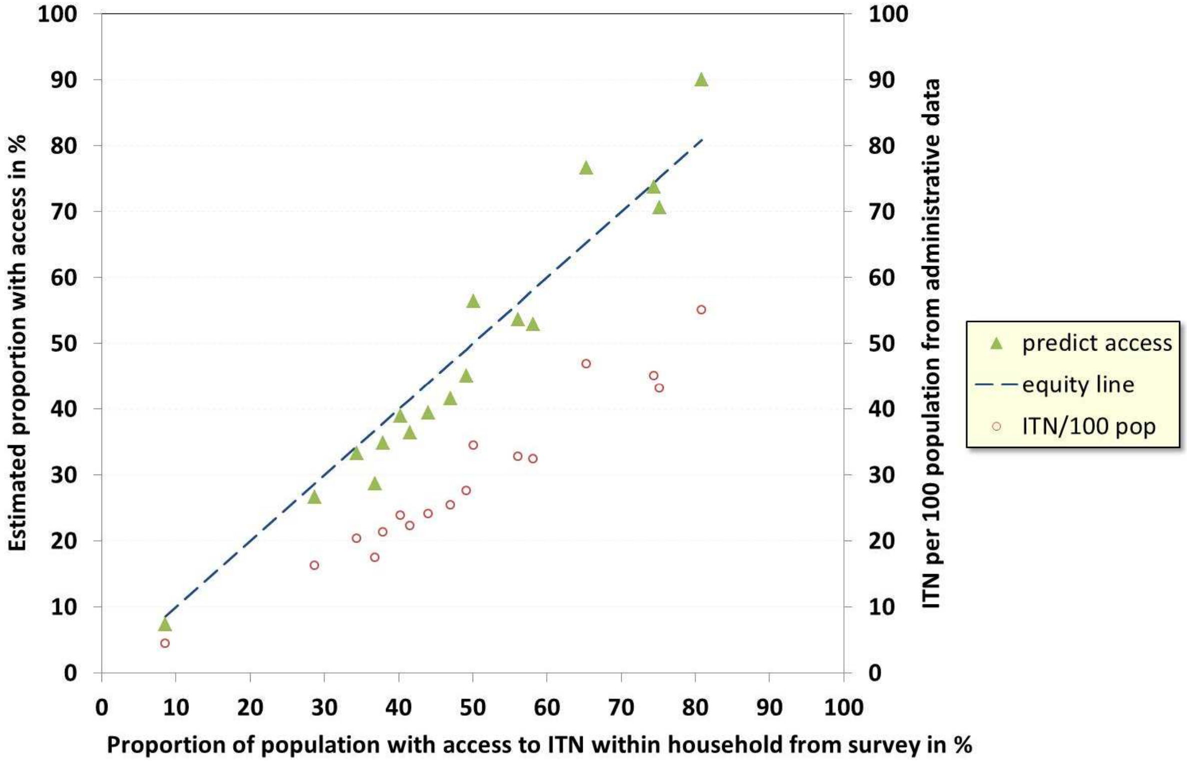

Box 3: Access coverage (c_a) of population with access to ITN within household

$$c_a = 1.6346 \left(\frac{n}{p} \times 100\right)$$

where
n= number of ITN in population
p= number of people in population

Net treatment and re-treatment

When NetCALC was created in 2005, net retreatments were prevalent and therefore a function was included in the model to retreat nets. However, with the availability of LLIN today, retreatment is no longer done with the exception of a few countries, such as Cambodia, where there are many untreated nets still being sold in the commercial markets. The main assumption for the net retreatment function is: if a net is conventionally dipped, it remains an ITN for only one year and then it moves into the compartment of untreated nets while it decays at the original rate of the cohort.

In a similar way the possibility of long-lasting net treatment was implemented (e.g. KO-Tab 123 or ICON MAXX). These nets will become and remain an LLIN as long as they are in the system as any other LLIN does, i.e. NetCALC does not turn LLIN into untreated nets even for the few that survive for five or more years. The main reason for this is that we do not really know at which point the insecticide content decreases below a point at which – provided the vectors are susceptible to pyrethroids – the LLIN ceases to provide additional protection to an untreated net. In addition, at an average net survival of three years, the contribution of LLIN older than five years is rather small and does not dramatically change the ITN coverage or access rate (see Figure 5).

Inputs needed

A detailed step-by-step explanation on the use of NetCALC is given in the user instruction file, but below is a brief overview over the inputs and how they are used in the calculations.
The basic inputs include:

1. The population size and the year of the last estimate
2. The growth rate
3. The average household size

With these inputs, NetCALC calculates for each year in the past (back to the year of the last net survey if that is before the last census) and future for a total of 30 years the total population and the number of households for each year. All functions, except the continuous distribution simulations, will run with this data.
However, if you want to test the potential contributions of different continuous distribution channels, you need more inputs because certain sub-populations attending health or education services have to be defined. For that you need:

1. The proportion of the population <5 years of age
2. The proportion under 15 years
3. The proportion of the total population pregnant per year

NetCALC will then construct an age-structured population matrix with annual cohorts from 1 to 20 years which allows extracting any given age sub-group to be calculated such children eligible for Expanded Program on Immunization (EPI), etc.

To actually calculate the people reached by certain service attendance or contact rates, the following is needed:

1. ANC attendance rates
2. EPI attendance rates

Initially only health service based distributions were included in NetCALC, however with an increasing interest to also include education services (such as school based distribution), the following information is needed:

1. The age bracket of children that are supposed to go to primary and secondary school. That varies from country to country. Some start with 6, some with 7, some have 6 years of primary school, others have 7 years of primary school.
2. The gross attendance ratio that is a standard educational indicator. It describes how many children are actually at school and is expressed as the number of school attending children over the number of children in the age bracket that should be going to school. In other words it includes those that are older and should have finished school but dropped out in between and have now come back. Therefore the rate often exceeds 100%. This is in contrast to the net attendance ratio, which only looks at the children in the school age bracket that are actually attending school. Since in a school distribution system a net would be given to all

students irrespective their age we need the gross attendance ratio to estimate the number of children that can be reached.

3. The third input is the dropout rate, i.e. the proportion of children that do not finish school out of those who started.

With this information NetCALC constructs a cohort for each school class that can be used for the projections the details of which are given in Box 4.

Box 4: Estimation of number of children attending school

Number of children in **first** year of primary/ secondary school (v_f)

$$v_f = \frac{v \times z}{w} + \left(\frac{v \times z}{w} - v_l\right)$$

Number of children in **last** year of primary/ secondary school (v_l)

$$v_l = \frac{v \times z}{w} \times (1 - z + u)$$

with

$$\mathrm{u} = 0.167795 + 0.4310174(\mathrm{z} - 0.5) - 0.4261722(z^3 - 0.125)$$

where

u=correction factor
v=number of children in school-going age
w=number of classes in school (primary or secondary)
y= gross attendance ratio (primary or secondary)
z= drop-out rate (primary or secondary)

Limitations

As a simple compartmental model the tool has a number of limitations one has to be aware of:

- The model does not exclude households that do not have children or pregnant women in them. For example, if you say that ANC attendance is X, Net CALC will calculate the number of pregnant women that can be reached and given a net. These nets will in the model then be distributed among all households and not only the sub-group with a pregnant woman attending ANC.
- NetCALC always assumes the net crop is distributed among all households in the population. The unit of allocation is not the individual household level, but rather the total of all households of the defined population in the district, region or country.
- It will not be able to take into account houses that are oversupplied. NetCALC will always allocate nets by the intra-household aggregation of nets on which the coverage formula is based. So if 30% of households are oversupplied and the others are undersupplied, the model cannot reflect this because it based on equitable distribution. Similarly, excess nets

that go beyond the 100% coverage in the defined population will not be taken out of circulation but will be “used” when the net crop begins to decline below the 100% level.
- In situations where a fixed number of nets are distributed to all households, irrespective of household size, NetCALC will under-estimate the “% with at least one ITN” and the deviation will be the higher the further the distributed number of nets is away from the 1 net per2 people quota.

In conclusion, NetCALC is only one element in the process of developing a comprehensive distribution strategy for ITN. It is to be used in connection with the guidelines on continuous distribution strategies that have been developed by the RBM Vector Control Working Group. NetCALC helps you to work in that process in thinking through the options of distribution. However, NetCALC cannot help with the political or operational decisions. It’s only once you have an assumption or a plan, that NetCALC can check whether these decisions look feasible? We can say, yes, if that is what you want, the numbers seem to fit. Go for it. Or we can say please, if you want to do that, it will not be sufficient to reach your targets. For that it has shown to be an excellent tool.
